# Key to *Syagrus* identification using leaflet margin anatomy: Supplement to “A revision of *Syagrus* (Arecaceae)”

**DOI:** 10.3897/phytokeys.81.12909

**Published:** 2017-06-15

**Authors:** Larry R. Noblick

**Affiliations:** 1 Montgomery Botanical Center, 11901 Old Cutler Road, Miami, Florida, USA 33156

**Keywords:** *Syagrus*, Arecaceae, anatomy, key, identification

## Abstract

Presented here is an alternative method of identification for species of the Neotropical palm genus *Syagrus*. It makes use of anatomical characters found in the leaflet margins and can be used for identification when few other vegetative or reproductive morphological characters are available. This anatomical study demonstrates the vast diversity found in a single palm genus and may also help to gain understanding of some possible relationships within the genus.

## Introduction

The following is meant to be a supplement to a revision of *Syagrus* (Noblick 2017). Palm leaflet anatomy has been useful in identification and has been used to suggest systematic relationships in the family. Tomlinson (1961) examined and described the leaflet anatomy of some 250 species of palms in 137 genera and suggested systematic relationships among genera. Horn et al. (2009) took it a step further and mapped lamina anatomy on the phylogenetic tree for the palm family based on plastid sequence data (Asmussen et al. 2006) in order to understand the evolution of lamina anatomy. Tomlinson’s brief survey (Tomlinson 1961) inspired Glassman to examine anatomy of the genus and its closely related genera in greater detail (Glassman 1972, 1987). Glassman (1972) emphasized that his survey of the genus was “based on mostly one collection for each taxon.” However by the time Glassman (1987) completed his revision, slides of two or more specimens were made for most taxa. His key was written as a convenient tool for identification and was not intended to show relationships. I produced a key based on leaflet anatomy to aid in the identification of 25 difficult to taxonomically distinguish *Syagrus* species with short, subterranean stems and was able to infer some relationships (Noblick 2013) that had been previously resolved by a molecular analysis (Meerow 2009). The key included herein has been completely rewritten and is meant to aid in the identification of all known 65 species and two subspecies of *Syagrus*.

Meerow et al. (2009) showed how leaflet anatomy supported the molecular relationships between *Allagoptera*, *Parajubaea* and *Polyandrococos* (now a synonym of *Allagoptera*).

Tomlinson et al. (2011) expanded his original 1961 work and presented information on 183 palm genera (out of 185 now recognized) and suggested relationships based on anatomy and the use of modern phylogenetic approaches.

Glassman (1972) emphasized various anatomical characters, several of which this paper makes use of, such as: (1) location of larger veins, (2) frequency and location (adaxial, abaxial, or in the middle) of intermediate and minor veins, (3) relative abundance, shape and location (adaxial and abaxial, or adaxial only) of clusters of nonvascular fibers, called fiber bundles (Tomlinson et al. 2011, Noblick 2013), (4) relative size of fiber bundles and veins at extremities of leaflets.

Tomlinson et al. (2011) only showed two *Syagrus* cross-sections in their publication: *S. orinocensis* and *S. weddelliana* (formerly *Lytocaryum*). However he suggested many anatomical features that vary in *Syagrus*, but the only ones used in this current study are: (1) the presence of trichomes, (2) the abundance and location of adaxial non-vascular fibers, varying from an almost continuous layer within the hypodermis to few fibers, (3) the extent and location of abaxial non-vascular fibers, (4) the extent to which the minor abaxial veins are in contact with the abaxial hypodermis, (5) the degree to which the inner sheath of major veins develop fibrous extensions to the upper surface layers.

## Materials and methods

### Plants examined

Both fresh and preserved (herbarium) material were used in this project. Dried material can be sectioned after rehydrating in a 5% solution of Contrad 70® (Decon Labs, King of Prussia, PA, U.S.A.) for a period of 24 hours (Tomlinson et al. 2011), though better results are obtained with fresh material.

The living material used in this study came mainly from the collections at Montgomery Botanical Center (MBC, Miami, FL) and the Jardim Botânico Plantarum (Nova Odessa, São Paulo, Brazil). A few were collected from Fairchild Tropical Botanic Garden (Coral Gables, FL). The dried material was often from air-dried specimens made while doing fieldwork, from the herbarium at Jardim Botânico Plantarum (HPL, Novo Odessa, São Paulo, Brazil), and from the Fairchild Tropical Botanic Garden herbarium (FTG, Miami, FL). A few specimens were from the following herbaria: G, IBGE, IPA, K, MO, NY and US.

### Anatomical preparation

**Figure 1.**
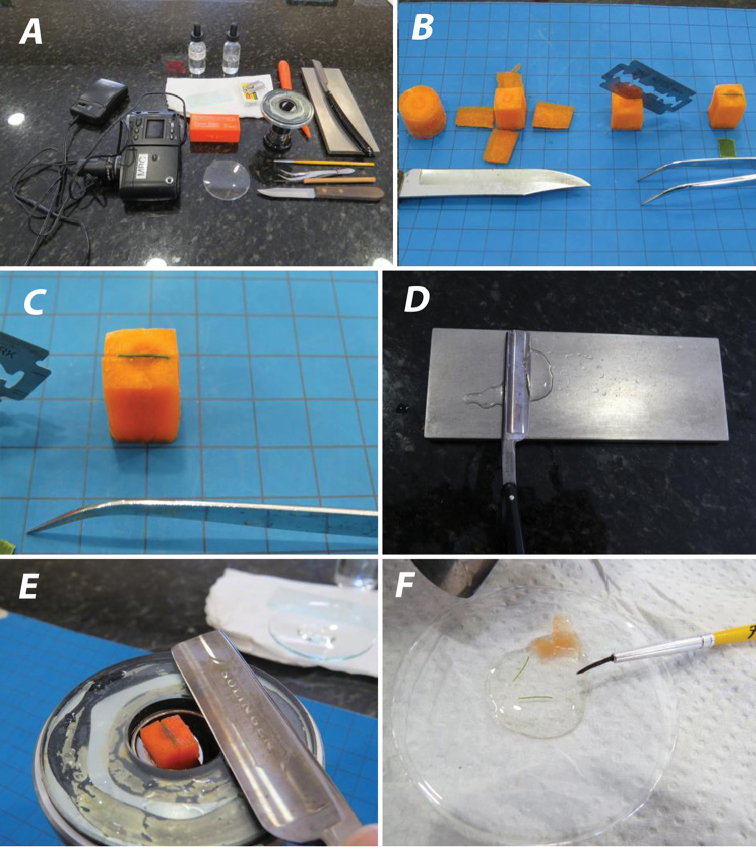
Materials and methods. **A** Materials used in making leaflet sections **B** Cutting carrot into a cube, slitting the cube and inserting the leaflet margin **C** Leaflet margin inserted in carrot cube ready for sectioning **D** Sharpening the straight razor on the wet stone **E** Clamping the carrot cube with leaflet margin into the hand microtome and preparing to section with the straight razor **F** Leaflet sections teased from the carrot into a watch glass with water.

There are a number of ways to hand section leaflet margins and most of these are covered in Noblick (2013). This type of research neither requires expensive hardware or use of chemicals and dyes. To start with, the leaflet sampled should always be collected from the same place on the plant. In this study, leaflets were sampled from the middle of a central leaflet. The following equipment was used: a hand microtome, a sharp knife, a straight razor, a double sided razor blade, a small artists brush (one of the smallest ones), a dropper bottle of water, a watch glass, a stone or plate to sharpen the straight razor blade, and a carrot (Figure 1A). The hand microtome was purchased from a home schooling site (Homesciencetools.com, Billings, MT, U.S.A.) for about $45. The traditional straight razor I use can be purchased from an online shaving store, though the one I purchased from no longer exists. One of the cheapest pre-sharpened stainless steel straight razors for beginners will probably do fine, and they can be purchased for as little as $14 from Jet.com (Hoboken, NJ, U.S.A.). I use a Dovo hollow ground stainless steel straight razor, which is available online for about $80. I prefer the traditional straight razor blade over those with replacement blades. Finally, to keep a razor-sharp edge, one needs a sharpening stone. The Dia-sharp 3 micron 8000 mesh (DMT D8EE 8” Extra Extra Fine Diamond Stone) (DMTsharp.com or Diamond Machining Technology, Marlbourough, MA, U.S.A) was found to be an excellent choice. Using it frequently between sectionings will maintain the sharpness required for making clean, thin sections.

### Preparing the section

Using a small sharp knife, a piece of carrot is cut into a small cube that will fit in the hand microtome (Figure 1B, C). A perpendicular, vertical slit is cut in the top of the cube with the double-sided razor blade, but not all the way through. A piece of the palm leaf margin is placed into the vertical carrot slit and the carrot cube is clamped into the hand microtome. The carrot cube is adjusted down until it is barely showing above the microtome plate. The first cut is made across the carrot to cut off the excess leaf material and carrot. The blade is re-sharpened using the Diamond Stone and water (Figure 1D). Lubricate the surface of the carrot with a drop of water and slide the straight razor across the microtome in a horizontal slicing movement, while pressing the side of the blade firmly against the plate (Figure 1E). If at first, it does not cut anything, adjust the hand microtome up about a ¼ of a turn and try again. Always keep the specimen lubricated with water using the eye dropper. After obtaining a section, tease the carrot away from the section or drop the section with attached carrot into a watch glass with water and tease the carrot from the section using the narrow artist brush, leaving the section in the watch glass (Figure 1F). After several sections have been successfully made, examine the sections in the watch glass under a dissecting microscope.

### Preparing the slide

To prepare the slide, some glass slides, glass cover slips, artist brush, a bottle of 1:1 glycerin and water solution, and a dissecting needle are needed. Glass slides with frosted glass along one side to write on are preferred. The glass slide and glass cover slip should always be cleaned with distilled water or 70 % alcohol before using. Label the frosted portion of the slide and spread a drop or two of the 1:1 glycerin and water solution on the slide. While looking through the dissecting microscope, select the thinnest sections from the watch glass with the narrow artist brush and place them into the 1:1 glycerin droplet on the slide. After placing a number of the sections on the slide (ca. 6), then cover the sections with the glass cover slip. The best way to insure that no bubbles are left behind under the cover slip is to use a dissecting needle. Place one edge of the cover slip at the edge of the glycerin droplet with the sections and begin gently lowering it into place over the sections. By placing the dissecting needle tip on its side under the other edge of the cover slip, while slowly pulling out the needle as the cover slip lowers into place, most of the air bubbles should exit from under the cover slip on the side of the exiting needle.

### Photography

The glass slide is now placed under a compound light microscope and photographed under the 10× objective (100× magnification). Images were taken with a Nikon Coolpix 4500 digital camera with a Leitz periplan 10 x/18 eye piece adapter. The camera was powered by a Powerline Universal AC adapter so there was no need to rely on battery power. The camera was set to P mode, white balance was adjusted to the light source of the microscope, and the lens was set to Fisheye 2. The images were cleaned of background spots, adjusted for brightness and contrast, and sharpened if necessary using Adobe Photoshop. Two adjacent images near the tip were photo merged using Adobe Photoshop. A stage micrometer was used to apply a scale to each image and replaced by a scale bar to aid in assembling these into a plate, while maintaining the scale.

### Characters defined

**Figure 2.**
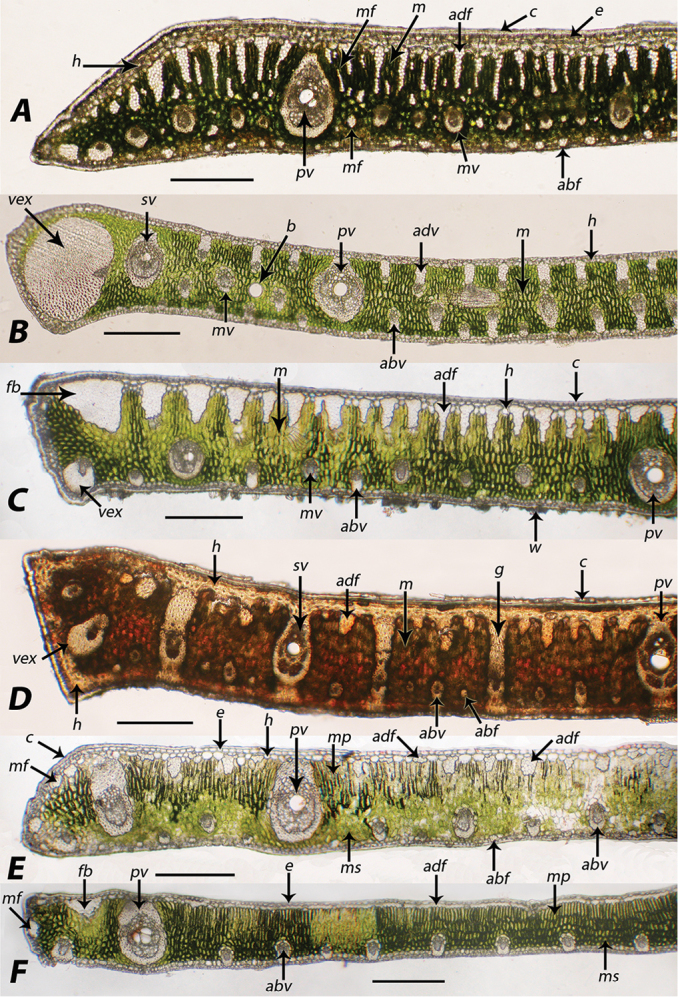
Characters. **A** *Syagrus vagans* with large adaxial fiber bundles reaching nearly ½ across the mesophyll, fiber bundles scattered throughout the mesophyll, mesophyll minor veins, abaxial fibers and a primary vein that is nearly connected to the hypodermis **B** *S. lilliputiana* showing a large marginal vein with an exaggerated fibrous sheath, adaxial minor veins, and an air bubble artifact **C** *S. coronata* with a large marginal fiber bundle, large adaxial fiber bundles, a small vein with an exaggerated fibrous sheath, and unattached primary vein **D** *S. harleyi* with a sclerenchymous hypodermis on the margin and along the adaxial surface, along with adaxial fiber bundles and abaxial minor vein symmetry (mirrored anatomy) and fibrous extensions from minor veins forming a girder **E** *S. orinocensis* with a mesophyll differentiated into palisade and spongy layers, marginal vein with fibrous sheath noticeably thickened adaxially, many fiber bundles along the adaxial surface, many of which are only one-cell thick and others reaching less than 1/5 across the mesophyll, also note very small abaxial fiber bundles often alternating with abaxial minor veins **F** *S. vermicularis* with an adaxial fiber bundle forming a continuous single layer of fiber cells in the hypodermis. **abf** = abaxial fiber bundle **adf** = adaxial fiber bundle **abv** = abaxial minor vein **adv** = adaxial minor vein **b** = trapped air bubble artifact **c** = cuticle **e** = epidermis **fb** = larger fiber bundle **g** = girder vein **h** = hypodermis **hf** = fibrous hypodermis **m** = mesophyll **mf** = marginal fiber bundle **mp** = palisade mesophyll **ms** = spongy mesophyll **mv** = mesophyll minor vein **pv** = primary vein **sv** = secondary vein **vex** = vein with exaggerated fibrous sheath **w** = wax. Bar scale = 0.25 mm.

This paper is focused mainly on characters of the more easily sectioned leaflet margin and not on the more difficult to section midrib. Epidermis and dark staining idioblasts (tanniniferous cells) were also not examined. Characters assessed during this study follow some of Glassman’s and Tomlinson’s characters as listed above in the introduction. Figure 2 clarifies much of the terminology for characters used in this paper. To be clear, in each leaflet cross-section the upper or superior side of the lamina is called the adaxial surface. The lower or inferior side is called the abaxial (Dransfield et al. 2008, Esau 1977).

The outer most layer of the leaf is the cuticle (Figure 2, c), a non-cellular waxy layer produced by the epidermis (Dransfield et al. 2008). The cuticle is followed by the epidermis, “outer skin” (Figure 2, e), followed by the hypodermis, “under skin” (Figure 2, h), which is finally followed by the mesophyll or “middle leaf” region (Figure 2, m). The mesophyll can be undifferentiated (m) or differentiated into a distinct palisade mesophyll (mp) and spongy mesophyll (ms). The palisade mesophyll near the adaxial surface is composed of vertically linear cells; the spongy mesophyll near the abaxial surface is composed of round cells and intercellular spaces of various sizes. Within the mesophyll are veins of various sizes, which are also known as vascular bundles or fibro vascular bundles (Tomlinson et al. 2011). In this paper they will be referred to as primary veins (Figure 2, pv), secondary veins (Figure 2, sv), and minor veins, which, depending on their location, are called mesophyll veins (Figure 2, mv), adaxial minor veins (Figure 2, adv) or abaxial minor veins (Figure 2, abv). Some primary and secondary veins are often attached to the adaxial hypodermis and sometimes to both adaxial and abaxial surfaces by fibrous sheath extensions. If the attachment extends to both surfaces via a narrow fibrous sheath extension, the vein appears girder-like and is indeed referred to as a girder (Tomlinson et al. 2011) (Figure 2D, g). In some veins the fibrous sheath becomes so enlarged with fibers that they are referred to as veins with exaggerated fibrous sheaths (Tomlinson et al. 2011) (Figure 2B, C, D, vex). Some veins have exaggerated sheaths both adaxially and abaxially as in *S. guimaraesensis* (Figure 5) or *S. pimentae* and *S. procumbens* (Figure 7). In addition to the veins, the laminal tissues are supported by nonvascular fibers or fiber bundles of various sizes. Some have large fiber bundles adjacent to or near their margins (Figure 2C, fb). Many fiber bundles are adaxial and may reach close to 1/3 to ½ or more across the mesophyll (Figure 2A, C, adf). Minor, intermediate and major (large) fiber bundles can be found adaxially (Figure 2A, C, D, E, adf). Many minor fiber bundles are mainly abundant abaxially (Figure 2A, C, D, E, abf). Occasionally minor fiber bundles are scattered throughout the mesophyll (Figure 2A, mf).

### Terminology defined

Mirrored anatomy—anatomy in which the abaxial surface is identical or similar to the abaxial surface. One surface appears to be reflected in the other. In this study, this term is not used exactly in the classical sense, where adaxial veins are exactly opposite other abaxial veins or adaxial fiber bundles are opposite abaxial fiber bundles (again, one surface reflected in the opposite surface). Here the term is used to describe the situation where structures are lined up perfectly opposite each other as if in a reflection. For example, adaxial fiber bundles may lie exactly opposite from abaxial veins or adaxial veins are opposite abaxial fiber bundles as in *S. mendanhensis* (Figure 6) or *S. pleioclada* (Figure 7). Again it is a reflection, but not necessarily of the same structures. This arrangement is not common in *Syagrus*.

Dorsiventral anatomy — anatomy in which the adaxial surface is very different from the abaxial surface. This is the anatomy most commonly seen in most *Syagrus* species.

### Characters utilized

The following characters were examined and used in this key:

**Veins**

• Presence/absence of adaxial minor veins

• Presence/absence of abaxial minor veins

• Presence of both adaxial and abaxial minor veins

• Quantity of adaxial minor veins (many, few)

• Adaxial minor veins (paired or opposite) abaxial minor veins

• Paired adaxial and abaxial minor veins (nearly touching or not)

• Presence/absence of abaxial vein with an exaggerated fibrous sheath

• Quantity of abaxial minor veins (many, few)

• Presence/absence of large marginal vein with exaggerated fibrous sheath

• Presence/absence of large marginal vein with exaggerated fibrous sheath on both adaxial and abaxial side

• Presence/absence of mesophyll minor veins

• Most mesophyll veins with or lacking abaxially thickened fibrous sheaths

• Location of mesophyll veins (middle, upper half, lower half)

• Presence/absence of primary or secondary vein (near or not near) the tip of the margin

• Primary, secondary or minor veins (attached, unattached)

• Surface to which the veins are attached (adaxial side only, both sides)

• Manner in which veins are attached (broadly attached, by one narrow fibrous extension, by two narrow fibrous extensions or girder-like)

• Order of arrangement of veins near the margin (secondary vein – minor veins –primary vein, primary vein – minor veins)

• Abaxial minor veins alternating with abaxial fiber bundles (or no such pattern).

**Fibers**

• Presence/absence of adaxial fiber bundles

• Size of the adaxial fiber bundles (reaching 1/3 to ½ across mesophyll, reaching less than 1/3, reaching less than 1/5 across mesophyll)

• Shape of adaxial fiber bundles (long and fat, long and skinny, elliptical, oblong, wedge-shaped, fat or skinny icicle-shaped, short and fat, irregular, rounded)

• First adaxial fiber bundle (the largest, ca. same size as others)

• Quantity of adaxial fiber bundles (many, occasional to none or few)

• Alignment of adaxial fiber bundles across from abaxial minor veins (aligned and opposite or not aligned)

• Mirrored versus dorsiventral anatomy (as defined above)

• Small adaxial fiber bundles alternating with larger adaxial fiber bundles

• Adaxial fiber bundles opposite abaxial veins (opposite each other, no such arrangement)

• Presence/absence of large to extra-large fiber bundle at or near the margin

• Presence/absence of abaxial fiber bundles

• Quantity of abaxial fiber bundles (many, few to none)

• Presence/absence of marginal minor fiber bundles (several, few to absent)

• Presence/absence of minor mesophyll fiber bundles

• Location of minor mesophyll fiber bundles (upper half, lower half, scattered throughout)

**Other non-fiber, non-vein characters**

• Differentiated or undifferentiated mesophyll

• If differentiated mesophyll, then the number of palisade layers (2 or 3 versus one)

• Cuticle layer (thick, thin)

• Adaxial hypodermis layer (sclerenchymous with thickened walls, near continuous one layer thickness of fibers, discontinuous layer of fibers, round bubble-like shaped cells, small rectangular shaped cells).

• Presence/absence of tiny fibers in the abaxial hypodermis layer

• Presence/absence of trichomes on the exterior abaxial surface

• Lamina thickness (<0.25 mm or 0.25 mm or more)

**Non-anatomical characters used in the key**

• Stem size (short underground [acaulescent], taller aerial stem)

• Stem number (solitary, in pairs or clustering)

• Leaf orientation (procumbent, spreading)

• Abaxial pubescence, tomentum or trichome color (brown, silvery) on the leaflets

• Leaf color (silver blue, green to dark blue-green)

• Middle leaflet measurements

• Leaf rachis length measurements

This key was designed for use in the field using simple tools and simple methods, which means using minimal equipment, no staining, low magnification (no higher than 100×), and the use of simple characters. Refer to the characters in the methods and Figure 2 for clarification. By using the methods listed above and following many of the simple techniques mentioned by Tomlinson et al. (2011), rapid results can be achieved in a laboratory provided with only the simplest of equipment.

## Results

This key contains headings, which will aid the reader to move through the key more rapidly. The headings refer back to the couplet that was responsible for bringing the reader to this section of the key. The number of the couplet that brought the reader to this section of the key is enclosed in parentheses following the heading. Besides aiding the reader to move more quickly through the key, the headings show the major subsections of the key. It is a way for the reader to understand where the major branches are in the key and which character separates species in that section of the key.

### Key

**Table:** 

1	Adaxial fiber bundles reach more than 1/3 to ½ across the mesophyll	**2**
–	Adaxial fiber bundles absent or reach less than 1/3 across the mesophyll	**37**
Adaxial fiber bundles large (1)		
2	No or few mesophyll minor veins (usually present only at the tip)	**3**
–	Mesophyll minor veins present (usually present throughout)	**18**
No or few mesophyll minor veins (2)		
3	Large marginal vein with exaggerated fibrous sheath occupying most of the margin	**4**
–	No such vein or vein with exaggerated fibrous sheath occupying less than ½ the margin	**10**
Large marginal vein with exaggerated sheath (3)		
4	Both abaxial and adaxial minor veins present	**5**
–	Only abaxial minor veins present and adaxial minor veins usually absent	**7**
5	Minor abaxial fiber bundles usually absent or if present not alternating with minor abaxial veins	***S. allagopteroides***
–	Minor abaxial fiber bundles present and alternating with abaxial minor veins	**6**
6	Adaxial minor veins common	***S. cerqueirana***
–	Adaxial minor veins occasional to none	***S. minor***
7	Mesophyll fibers or fiber bundles restricted to the upper half of the mesophyll	***S. campestris***
–	Mesophyll fibers or fiber bundles absent in the upper half of the mesophyll	**8**
8	Adaxial fiber bundles linear, like skinny icicles	***S. romanzoffiana***
–	Adaxial fiber bundles fat and elliptical or oblong	9
9	Large adaxial fiber bundles abundant	***S. cataphracta***
–	Larger adaxial fiber bundles occasional	***S. deflexa***
No large marginal vein with exaggerated sheath (3')		
10	Adaxial and abaxial minor veins present	**11**
–	Adaxial minor veins absent	**12**
11	Adaxial minor veins many and usually attached by a large fibrous extension	***S. cerqueirana***
–	Adaxial minor veins few and attached by a relatively small fibrous extension	***S. yungasensis***
12	Lamina thin, less than 0.25 mm thick	***S. macrocarpa***
–	Lamina thicker, 0.25 mm or thicker	**13**
13	Primary vein attached to adaxial but not abaxial surface	**14**
–	Primary vein attached to both surfaces	**15**
14	Large to medium-sized first adaxial fiber bundle near the margin, most other adaxial fiber bundles elliptical or oblong in shape	***S. lorenzoniorum***
–	Very large first adaxial fiber bundle near the margin, most other adaxial fiber bundles wedge-shaped, like fat icicles	***S. caerulescen***
15	Secondary and minor veins attached to both surfaces forming narrow ‘girders’	***S. campylospatha***
–	Minor veins attached to the abaxial surface only, not forming narrow ‘girders’	**16**
16	Small adaxial fiber bundles alternating with larger adaxial fiber bundles	**S. graminifolia subsp. graminifolia**
–	Fiber bundles all the same size	**17**
17	Secondary vein with a short narrowing adaxial fibrous extension like a vase	**S. graminifolia subsp. cabraliensis**
–	Secondary vein with longer narrow adaxial fibrous extension like a wine bottle	**S. graminifolia subsp. glazioviana**
Mesophyll minor veins present (2')		
18	Scattered mesophyll fibers or fiber bundles absent	**19**
–	Scattered mesophyll fibers or fiber bundles present	**32**
No Mesophyll fibers (18)		
19	Abaxial fibers, abaxial fiber bundles or abaxial minor veins none or only weakly visible, abaxial veins present with little abaxial thickening and alternating with larger mesophyll veins	**20**
–	Many Abaxial fiber, abaxial fiber bundles and/or abaxial minor veins present and obvious, minor abaxial veins usually thickened abaxially and not alternating	**27**
20	Adaxial fiber bundles opposite abaxial minor veins, mirrored anatomy	**21**
–	Adaxial fiber bundles and abaxial minor veins not necessarily opposite each other, dorsiventral anatomy	**22**
21	One minor adaxial fiber bundle-abaxial minor vein pair between primary and secondary veins	***S. mendanhensis***
–	Three minor adaxial fiber bundle-abaxial minor vein pairs between primary and secondary veins	***S. pleioclada***
22	Weakly visible abaxial veins alternating with larger mesophyll veins	*S. comosa*
–	No abaxial veins present	**23**
23	No extra-large adaxial fiber bundles on or near the margin	***S. gouveiana***
–	Extra-large adaxial fiber bundles on or near the margin	**24**
24	Primary veins attached to the adaxial surface	**25**
–	Primary veins unattached	**26**
25	Hypodermal cells rounded or bubble-like, especially between the adaxial fiber bundles, no tiny fibers in the abaxial hypodermis	***S. duartei***
–	Hypodermal cells smaller, rectangular, especially near the adaxial fiber bundles, small, almost indistinguishable fibers in the abaxial hypodermis	***S. evansiana***
26	Adaxial fiber bundles short and fat, irregular-shaped to rounded	***S. kellyana***
–	Adaxial fiber bundles long and fat, mostly wedge-shaped, like skinny or fat icicles	***S. coronata***
27	Marginal hypodermis with thick sclerenchymous walls, secondary and tertiary veins attached to the adaxial surface via long narrow fibrous sheath extensions	***S. harleyi*** (high elevation form)
–	Marginal hypodermis without sclerenchymous walls, secondary and tertiary veins unattached or fibrous sheath extensions wider than 2 or 3 cells	**28**
28	Abaxial minor veins clearly alternating with abaxial fiber bundles	***S. petraea***
–	Mostly abaxial minor veins along the abaxial surface, abaxial fiber bundles infrequent and not alternating as above	**29**
29	Palm develops an aerial stem	***S. glaucescens***
–	Palm develops only short, underground stem	**30**
30	Few (3–8) attached abaxial minor veins and abaxial fiber bundles between larger veins, sometimes with a large fiber bundle near the margin, but frequently lacking	***S. glazioviana***
–	Many (9–12+) abaxial minor veins and abaxial fiber bundles between larger veins, always with a large fiber bundle near the margin	**31**
31	Primary veins attached, silvery-blue leaves with middle leaflets 24–34 × 2–3 cm	***S. rupicola***
–	Primary veins unattached, gray-blue leaves with shorter, narrower middle leaflets 10-20 × 0.7–1.2 cm	***S. longipedunculata***
Mesophyll fibers present (18')		
32	Abaxial fiber bundles or fibers absent or nearly so or not readily apparent	**33**
–	Abaxial fiber bundles or fibers present and very obvious	**34**
33	First adaxial fiber bundle the largest	***S. microphylla***
–	First adaxial fiber bundle smaller or not much larger than the second	***S. werdermannii***
34	Primary veins clearly attached and some of the first adaxial fiber bundles larger, elliptical to rounded and oblong and the rest like fat icicles, mesophyll fibers few	***S. ruschiana***
–	Primary veins clearly unattached or nearly so, first adaxial fiber bundles smaller or more angular in shape and the rest like skinny icicles, mesophyll fibers more abundant	**35**
35	Primary veins sometimes nearly attached	***S. vagans***
–	Primary veins obviously unattached	**36**
36	Large fiber bundle and veins with exaggerated fibrous sheaths near the margin	***S. santosii***
–	Large fiber bundle absent, adaxial minor veins sometimes present near the margin, much smaller marginal minor veins with exaggerated fibrous sheath present	***S. schizophylla***
Adaxial fiber bundles small to none (1')		
37	Mesophyll minor veins present or at least several near the margin	**38**
–	Mesophyll minor veins absent	**46**
Mesophyll minor veins present (37)		
38	Very large marginal vein with exaggerated fibrous sheath adaxially and abaxially	**39**
–	No such marginal vein	**40**
39	Most mesophyll veins with abaxially thickened fibrous sheaths, medium to dark green leaves procumbent, leaf rachis 2.4–13 cm long	***S. procumbens***
–	Most mesophyll veins lacking abaxially thickened fibrous sheaths, silvery bluish-green leaves spreading, leaf rachis 44–65 cm long	***S. pimentae***
40	Minor adaxial veins present	**41**
–	Minor adaxial veins absent	**42**
41	Large marginal vein with exaggerated fibrous sheath present	***S. lilliputiana***
–	No such large marginal vein present, but a large vein with thickened sheath is present a short distance from the margin	***S. guimaraesensis***
42	Few to no adaxial fiber bundles present, lamina less than 0.25 mm thick	***S. pompeoi***
–	Adaxial fiber bundles always present even if small, lamina thicker than 0.25 mm	**43**
43	Mesophyll minor veins located in the mid to upper half of the mesophyll, mesophyll undifferentiated, short palm with an underground stem	***S. pleiocladoides***
–	Mesophyll minor veins located in the lower half of the mesophyll, the latter differentiated into palisade and spongy mesophyll cells, palms with aerial columnar stems	**44**
44	Cuticle layer, very thick, first adaxial fiber bundle the largest	***S. kellyana***
–	Cuticle layer, very thin, not obvious, first adaxial fiber bundle not always the largest	**45**
45	Large primary vein near the margin, stems solitary	***S. oleracea***
–	Only secondary or minor veins near the margins, stems often in pairs or clustering	***S. cearensis***
No Mesophyll minor veins (37')		
46	Large marginal vein with exaggerated fibrous sheath	**47**
–	No such marginal vein present	**51**
Large marginal vein with exaggerated sheath (46)		
47	Mesophyll fibers present in the upper half of the mesophyll	***S. itacambirana***
–	No mesophyll fibers present	**48**
48	No adaxial minor veins	***S. loefgrenii***
–	Adaxial minor veins present and often paired with abaxial minor veins	**49**
49	Adaxial minor veins and abaxial minor veins paired and opposite each other, but never nearly touching	***S. angustifolia***
–	Several minor adaxial veins paired and nearly touching abaxial minor veins	**50**
50	A few adaxial and marginal minor fiber bundles present	***S. menzeliana***
–	Adaxial and marginal minor fiber bundles absent	***S. emasensis***
No large marginal vein with exaggerated sheath (46')		
51	Hypodermis with thickened sclerenchymous walls especially on the margin, adaxial fiber bundles are usually opposite abaxial minor veins	**52**
–	Hypodermis lacking thick sclerenchymous walls, adaxial fiber bundles not usually opposite adaxial minor veins	**53**
52	Sclerenchymous hypodermis continues along the adaxial surface, all adaxial minor veins are usually attached	***S. harleyi*** (low elevation form)
–	Sclerenchymous hypodermis only on the margin, every other abaxial minor vein (mesophyll vein) is unattached	***S. harleyi*** (high elevation form)
53	Most veins attached to both surfaces of the leaflet and/or leaflet with hairy trichomes on the abaxial surface	**54**
–	Most veins attached to either the adaxial or abaxial surface but not to both and lacking hairy trichomes	**57**
54	Most veins attached only to the abaxial surface or attached adaxially only by a very short fibrous extension, palm with short underground stem	***S. itapebiensis***
–	Most veins attached to both surfaces and to the adaxial surface by a very narrow fibrous extension, palm with aerial stem	**55**
55	Mesophyll differentiated into palisade and spongy mesophyll cells	***S. weddelliana***
–	Mesophyll undifferentiated	**56**
56	Leaf rachis 140–155 cm, leaflet with brownish pubescence abaxially	***S. hoehnei***
–	Leaf rachis less than 99 cm, leaflet with silvery pubescence abaxially	***S. insignis***
57	Mesophyll undifferentiated	**58**
–	Mesophyll differentiated	**60**
58	Primary veins clearly unattached	***S. picrophylla***
–	Primary veins attached adaxially or nearly so	**59**
59	First fiber bundle the largest, leaf rachis ca. 100 cm long	***S. elata***
–	First fiber bundle ca. same size as the others, leaf rachis 167–440 cm long	***S. romanzoffiana***
60	Two or three layers of palisade mesophyll cells on the adaxial surface, lamina usually 0.25 mm thick or more	**61**
–	One layer of palisade mesophyll cells on the adaxial surface, lamina usually less than 0.25 mm thick	**70**
61	Abaxial fiber bundles alternating with abaxial minor veins	**62**
–	Abaxial fiber bundles absent or with only an occasional one present	**67**
62	Large vein near the tip of the margin	**63**
–	No large vein near the tip of the margin	**65**
63	Marginal vein with fibrous sheath not noticeably thickened and adaxial fiber bundles very obvious	***S. amara***
–	Marginal vein with fibrous sheath noticeably thickened, abaxial fiber bundles very small and sometimes difficult to distinguish	**64**
64	Marginal vein with fibrous sheath noticeably thickened both abaxially and adaxially	***S. stenopetala***
–	Marginal vein with fibrous sheath noticeably thickened only adaxially	***S. orinocensis***
65	Marginal fiber bundles not present or not very large	***S. stratincola***
–	Marginal fiber bundles fairly large, especially on the adaxial surface	**66**
66	A nearly continuous single layer of fiber cells in the adaxial hypodermis and fiber bundles along the adaxial surface, Amazonas	***S. inajai***
–	Occasional fiber cells in the adaxial hypodermis and fiber bundles often lacking, Atlantic Forest	***S. botryophora***
67	Secondary vein near the tip, followed by several minor veins and the primary vein	**68**
–	Primary vein near the tip, followed by several minor veins	**69**
68	Many fibers or fiber bundles along the adaxial surface, adaxial vein with an exaggerated fibrous sheath absent or very small	***S. cocoides***
–	No or very few fibers or fiber bundles along the abaxial surface, an abaxial vein with an exaggerated fibrous sheath is often present near the margin	***S. flexuosa***
69	Nearly continuous one layer of fibers in the adaxial hypodermis	***S. vermicularis***
–	Interrupted layer of fibers and fiber bundles in the adaxial hypodermis	***S. pseudococos***
70	Primary vein unattached, abaxial fiber bundles appearing flattened	***S. smithii***
–	Primary vein attached adaxially, abaxial fiber or fiber bundles, if present, small and rounded	**71**
71	Adaxial fibers and fiber bundles form a continuous layer in the hypodermis, abaxial fiber fibers and fiber bundles occasionally present	***S. sancona***
–	Adaxial fibers and fiber bundles forming a discontinuous layer in the hypodermis, abaxial fibers and fiber bundles absent	***S. cardenasii***

## Discussion

### Variation

**Figure 3.**
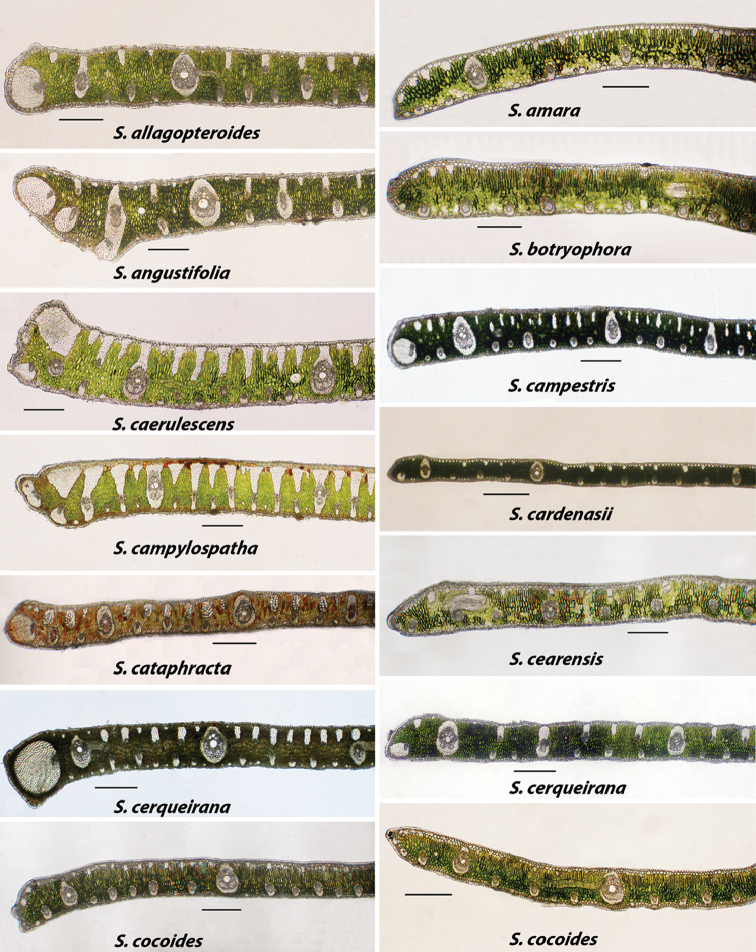
Alphabetically arranged marginal leaflet transections. *Syagrus allagopteroides* to *S. cocoides*. Bar scale = 0.25 mm.

**Figure 4.**
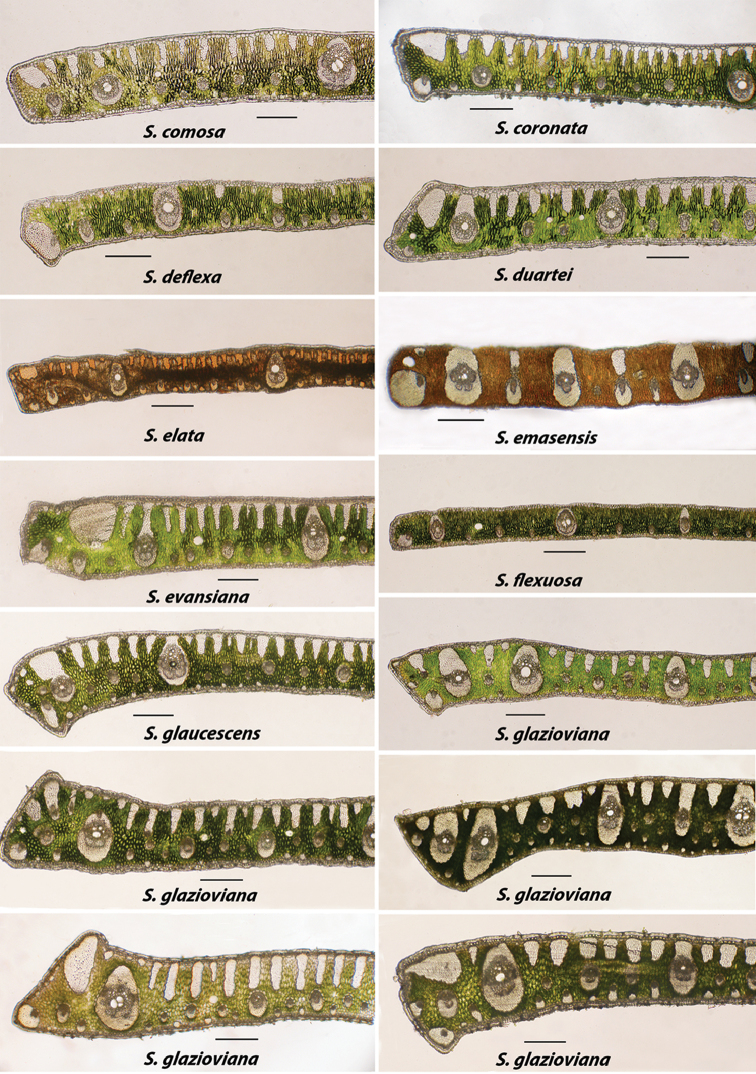
Alphabetically arranged marginal leaflet transections. *Syagrus comosa* to *S. glazioviana*. Note variation in *S. glazioviana*. Bar scale = 0.25 mm.

**Figure 5.**
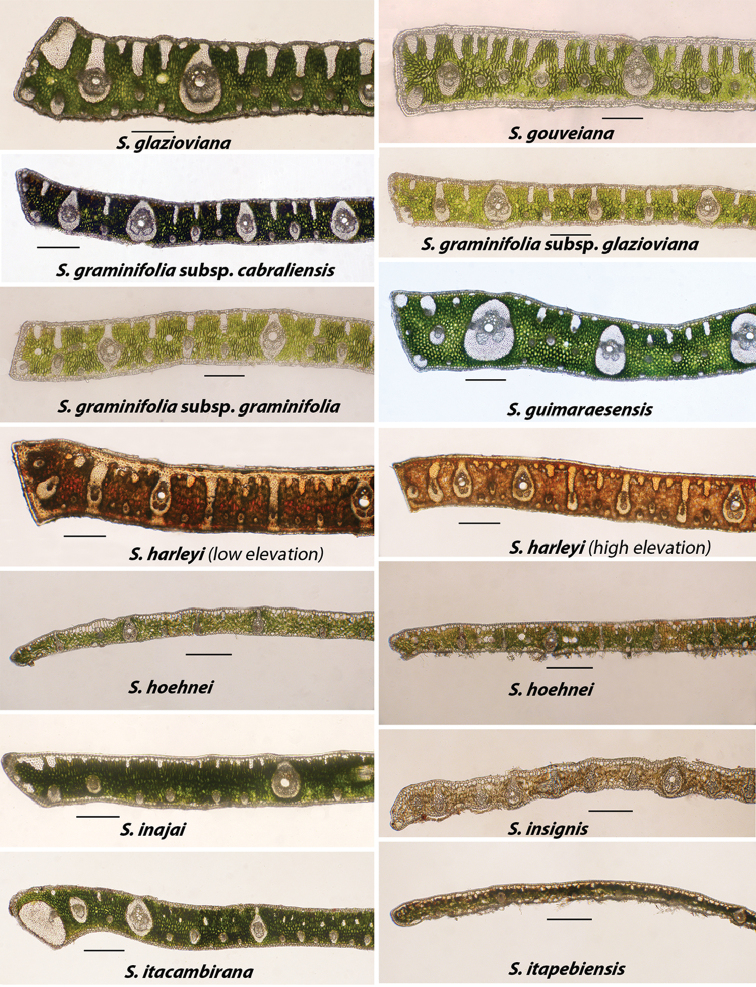
Alphabetically arranged marginal leaflet transections. *Syagrus glazioviana* to *S. itapebiensis*. Note variation in *S. harleyi* and *S. hoehnei*. Bar scale = 0.25 mm.

I have examined several specimens for each species. However, I make no claim that this has been an exhaustive study. In making use of this key the user must allow for slight variation due to environmental and population differences from the published images. As more specimens were sampled, I expected to see more variation, but was surprised to see how many species stayed true to their basic arrangement of veins and fiber bundles, as well as agreed with previous work done by Glassman (1972, 1987). Nonetheless, a few do vary and for this reason more than one image is used to represent some species. Examples of this are seen in *Syagrus cerqueirana* (Figure 3) where the marginal vein with the exaggerated fibrous sheath varies in size along with the number of adaxial and abaxial minor veins and fiber bundles. Another example is *S. cocoides* (Figure 3), where the marginal tip shape varies, along with the quantity and frequency of adaxial fiber bundles. *Syagrus glazioviana* (Figure 4, 5) is an extreme example of variation. The variation seen in this species either represents true variation within a single species or it is revealing a complex of several closely related species. Environment definitely plays a part in this variation as seen in *S. harleyi* (Figure 5) where the low elevation form has a sclerenchymous hypodermis on the margins and along the adaxial surface making it more drought resistant, while the high elevation form has a sclerenchymous margin, but has less sclerenchymous tissue along the adaxial surface. The variation seen in *S. hoehnei* (Figure 5) represents two different populations (Harri Lorenzi, pers. comm.). *Syagrus minor* also represents two specimens separated by over 100 km with one having adaxial veins, which are nearly absent in the other. The variation seen in *Syagrus vagans* (Figure 8) clearly demonstrates the futility of trying to use marginal shape for species determination, at least in *Syagrus*.

**Figure 6.**
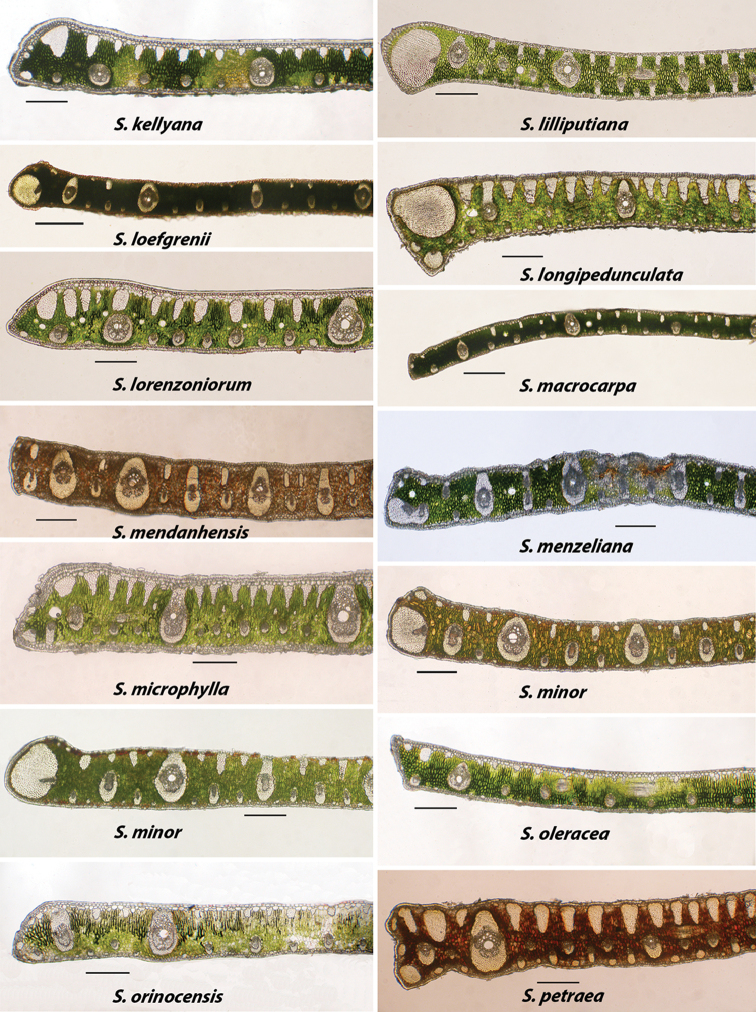
Alphabetically arranged marginal leaflet transections. *Syagrus kellyana* to *S. petraea*. Note variation in *S. minor*. Bar scale = 0.25 mm.

**Figure 7.**
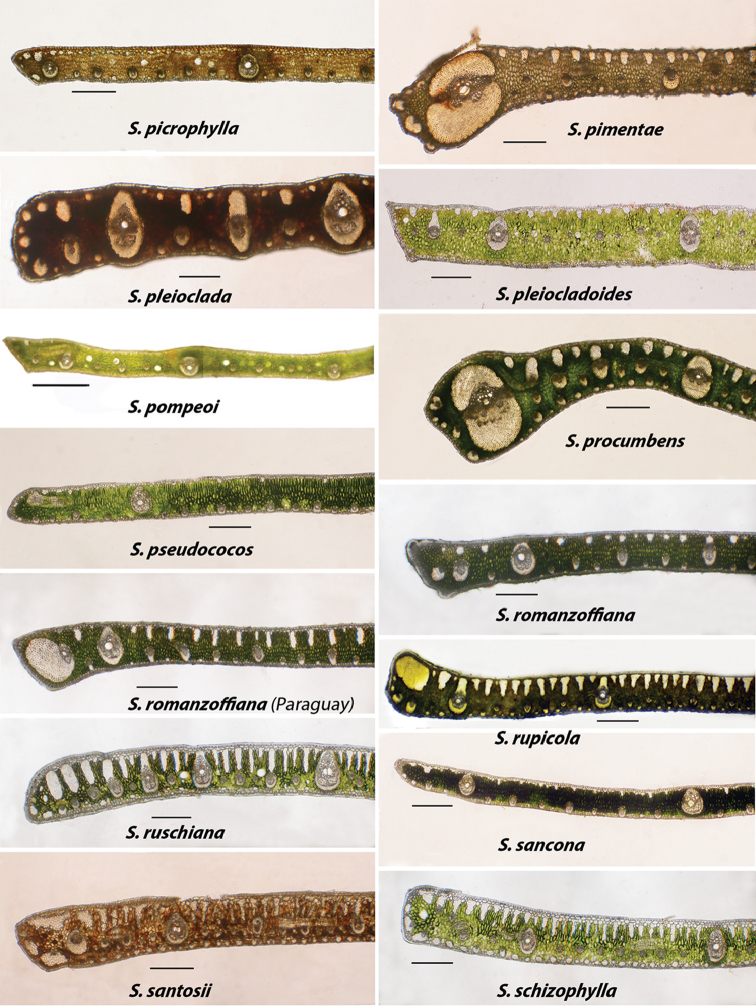
Alphabetically arranged marginal leaflet transections. *Syagrus picrophylla* to *S. schizophylla*. Note variation in *S. romanzoffiana*. Bar scale = 0.25 mm.

### Molecular clades versus phenetic (key) branch similarities

Although I was not attempting to show close relationships in his key, nevertheless, certain relationships are suggested based on how these species resolved in the key. Such relationships can only be confirmed further by an in depth phylogenetic molecular analysis of the genus.

An outline of the key is presented (Figure 9, 10) to allow one to more clearly visualize the branches of the key and is not meant to depict a phylogenetic analysis. This is not a cladogram. An interesting, but unintentional consequence of producing this key has been to discover how species might be related based on their anatomical phenetics, i.e. possible relationships based on their overall similarity in anatomy or the organization of their anatomical characters. It must be emphasized that this is not an actual phenetic analysis using some kind of distance coefficient. In some cases, branches of the key contain species that had been previously shown to be related by molecular analyses (Meerow et al. 2009, 2014). As demonstrated in Table 1, species of the former genus *Lytocaryum* all resolve in the same branch of the key (Figure 10, branch 53) as they did in the molecular analysis of those analyzed. Species of the strongly supported Rain Forest clade (Meerow 2009, 2014) all emerge together in a branch of the key containing mostly Amazonian, Caribbean, Andean and few Atlantic forest species (Figure 10, branch 60). The Eastern Brazilian species of the molecular analyses are not as neatly grouped, but of the few species analyzed, they emerge in two portions of the key, but usually among other Eastern or Central-Western Brazilian species (Table 1, Figure 9, branches 18 and 43’). Cluster stemmed species mostly emerge in the same branch of the key (Table 1, Figure 9, branch 3’).

**Figure 8.**
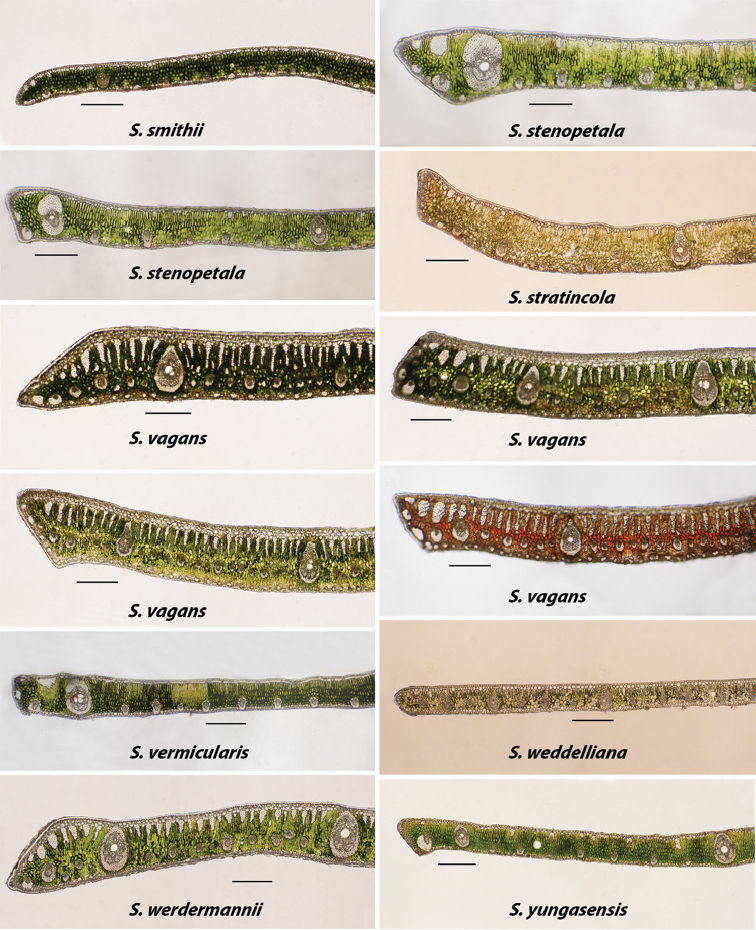
Alphabetically arranged marginal leaflet transections. *Syagrus smithii* to *S. yungasensis*. Note variation in *S. stenopetala* and *S. vagans*. Bar scale = 0.25 mm.

**Figure 9.**
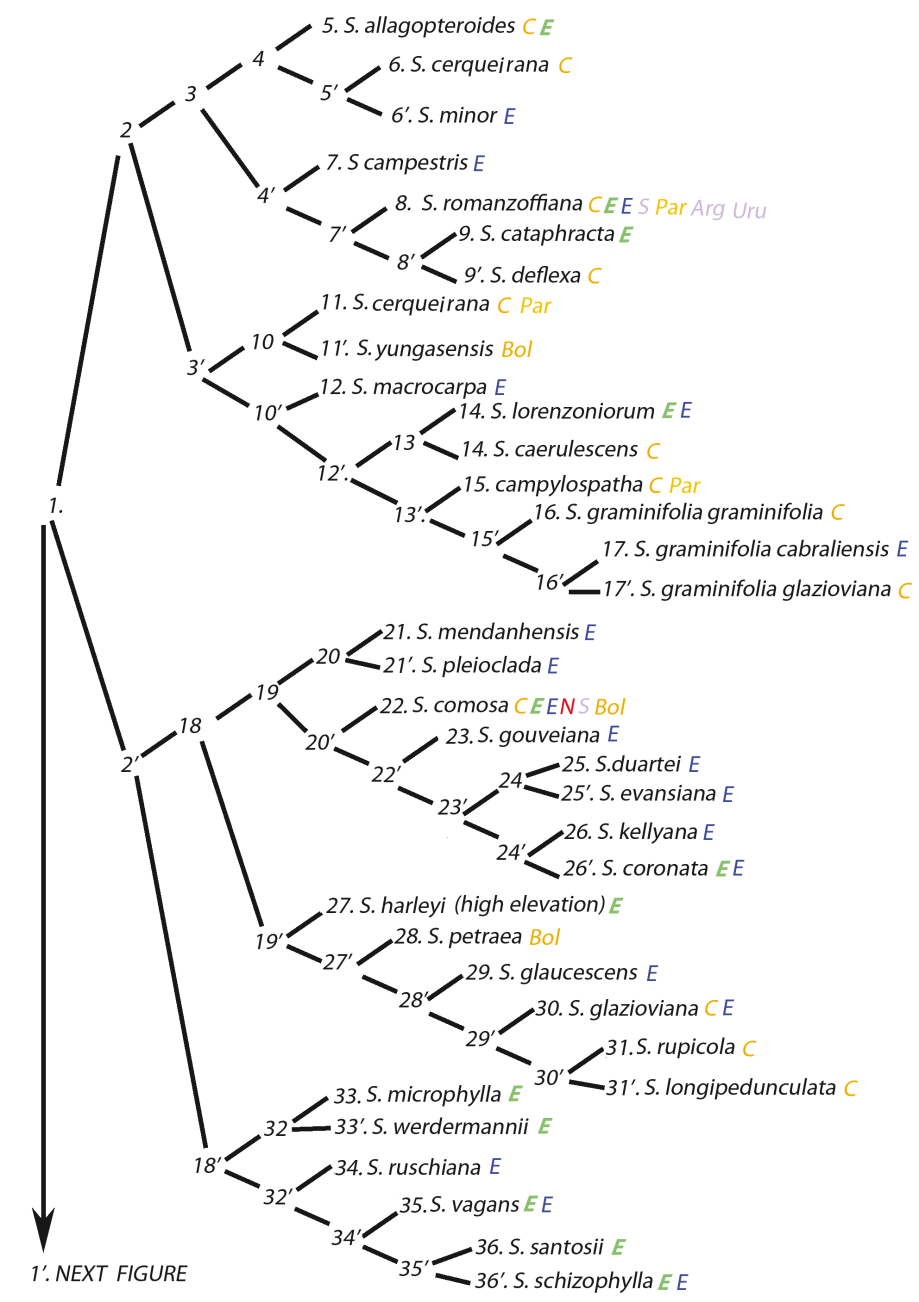
Diagram of the anatomical key to *Syagrus*. Branch 1. Colored letters represent regions of distribution for that species. For regions of Brazil: **Orange C** = Central-West **Green bold italic E** = Northeast **Blue E** = Southeast **Red N** = North and **Violet S** = South. For other countries: **Arg** = Argentina **Bol** = Bolivia **Par** = Paraguay and **Uru** = Uruguay.

**Figure 10.**
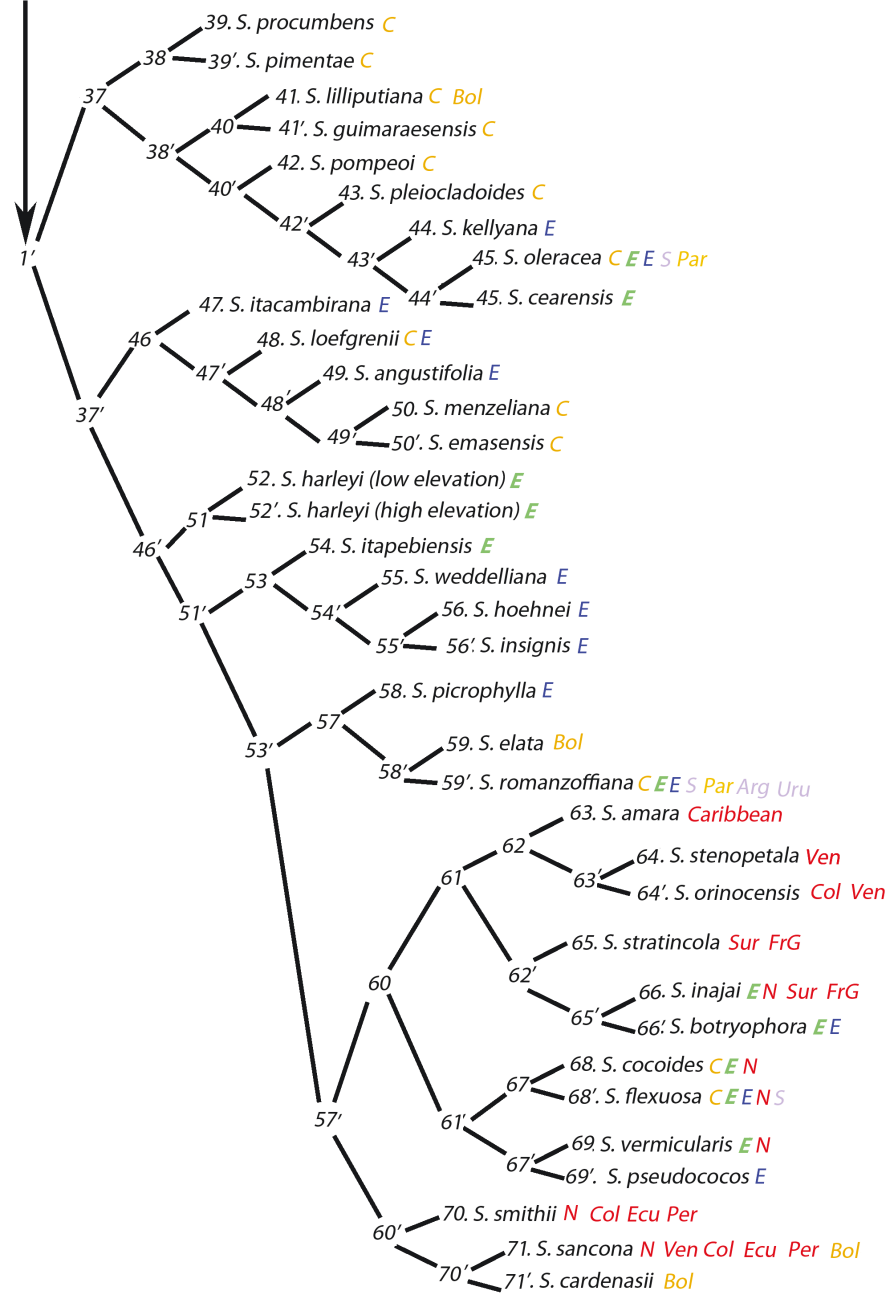
Continuation of the diagram of the anatomical key to *Syagrus*. Branch 1’. Colored letters represent regions of distribution for that species. For regions of Brazil: **Orange C** = Central-West **Green bold italic E** = Northeast **Blue E** = Southeast **Red N** = North and **Violet S** = South. For other countries: **Arg** = Argentina **Bol** = Bolivia **Col** = Colombia **Ecu** = Ecuador **FrG** = French Guyana **Par** = Paraguay **Per** = Peru **Sur** = Surinam **Uru** = Uruguay and **Ven** = Venezuela.

**Table 1.** Comparison of molecular clades and key branches. Meerow et al. (2009) designated three clades based on nuclear DNA sequences in *Syagrus*: Rain Forest, Eastern Brazilian, and Cluster stemmed. Taxa with an asterix (*) in column 4 were part of the original molecular analysis (Meerow et al. 2009).

Name of Clade or Region	Molecular Clade (Meerow 2009)	Key Branch	Species found in similar Branch of Key	Geographical Region
Rain Forest/ *Lytocaryum*	*L. itapebiensis L. weddelliana*	53	S. itapebiensis* S. weddelliana* S. hoehneiS. insignis	Atlantic Forest Atlantic Forest Atlantic Forest Atlantic Forest
Rain Forest	*S. amaraS. orinocensisS. stenopetalaS. botryophoraS. cocoidesS. vermicularisS. sanconaS. ruschiana*	57'	S. amara* S. orinocensis* S. stenopetala* S. inajaiS. botryophora* S. stratincolaS. cocoides* S. flexuosa* S. vermicularis* S. pseudococosS. smithiiS. sancona* S. cardenasii	Caribbean Amazon Amazon Amazon Atlantic Forest Amazon Amazon Central Brazil Amazon Atlantic Forest Amazon Andes/Amazon Andes
Eastern Brazilian	*S. cearensisS. coronataS. glaucescensS. kellyanaS. oleracea*	18	S. mendanhensisS. pleiocladaS. comosaS. gouveianaS. duarteiS. kellyana* S. coronata* S. harleyiS. petraeaS. glaucescens* S. glaziovianaS. rupicolaS. longipedunculata	Eastern Brazil (MG) Eastern Brazil (MG) E. & Central Brazil Eastern Brazil (MG) Eastern Brazil (MG) Eastern Brazil (MG) Eastern Brazil Eastern Brazil (BA) Bolivia Eastern Brazil (MG) E. & Central Brazil Central Brazil (GO) Central Brazil (GO)
Eastern Brazilian		43'	S. kellyana* S. oleracea* S. cearensis*	Eastern Brazil E. & Central Brazil Eastern Brazil
Clustered stemmed	*S. campylospathaS. cerqueiranaS. macrocarpaS. flexuosa*	3'	S. cerqueirana* S. yungasensisS. macrocarpa* S. lorenzoniorum S. caerulescens S. campylospatha* S. graminifolia	C. Brazil /Paraguay Andes (Bolivia) Eastern Brazil (MG) Eastern Brazil (MG) Central Brazil (GO) C. Brazil /Paraguay C. Brazil (GO, MG)

### Species from the same or neighboring geographic regions

Frequently, neighboring species key out in the same branch (Figure 9, 10). Branch 2 contains species primarily from the Central-West region of Brazil. *Syagrus allagopteroides* and *S. minor* are from adjacent areas of Goiás and Minas Gerais (branch 4). All of the three subspecies of *S. graminifolia* (as defined by Noblick, 2017) fall out together in the key (branch 15’) and while they are from two different geographic regions of Brazil (Southeast and Central-west), they are really only from the neighboring states of Goiás and Minas Gerais, which just happen to form the boundary between these two regions.

Branch 19 contains species that can all be found in the state of Minas Gerais, Brazil. *Syagrus mendanhensis* and *S. pleioclada* (branch 20) are from the neighboring areas of the Serra Diamantina and Serra do Cipó in Minas Gerais as are *S. gouveiana*, *S. duartei*, and *S. evansiana* (branch 19). In that same branch, *Syagrus kellyana* grows in the neighboring Serra do Mar region of Minas Gerais closer to the coast, while *S. coronata* can be found growing in the northeastern part of the same state adjacent to its Bahian distribution.

Species of branch 19’, with one exception, come from the Central-West or adjacent Southeastern region of Brazil. *Syagrus glazioviana*, *S. rupicola* and *S. longipedunculata* (branch 29’) can all be found in the state of Goiás with *S. rupicola* and *S. longipedunculata* restricted to the Serra dos Veadeiros in the northern part of Goiás while *S. glazioviana* is more widespread.

Several Bahian species emerge in branch 18’. *Syagrus microphylla* and *S. werdermannii* grow in the mountainous interior of Bahia. *Syagrus vagans* tolerates the dry caatinga habitat in and between the mountains and the coast of Bahia. Finally, the rare, recently discovered *S. santosii* and more common *S. schizophylla* flourish near the coast.

With few exceptions most of the species in branch 37 grow in Brazil’s Central-West region, Mato Grosso, Mato Grosso do Sul, and Goiás (*S. procumbens*, *S. pimentae*, *S. lilliputiana*, *S. guimaraesensis*, *S. pompeoi*, *S. pleiocladoides*, *S. oleracea*). However, the *S. oleracea* distribution overlaps with *S. cearensis* from Brazil’s Northeast region and at one time they were considered to be the same species (Glassman 1987, Noblick 2004, 2017).

Branch 46 contains cerrado species from the adjacent Central-West and Southeast regions of Brazil. *Syagrus emasensis* and *S. menzeliana* are neighboring species (branch 49’) with the first growing in the southwestern corner of Goiás in the Parque Nacional das Emas and the other growing just south of the park.

It is not surprising to see both forms of *S. harleyi* key out in branch 51. However, the high elevation form also may key out earlier in branch 19 as some of the abaxial minor veins can be interpreted as being mesophyll veins.

All of the former *Lytocaryum* species (branch 53) grow in Atlantic Forest of Eastern Brazil from Brazil’s Northeast region and the eastern hills of Bahia (*S. itapebiensis*), through Brazil’s Southeast region including the Serra do Mar mountains of Espirito Santo (*S. insignis*), Rio de Janeiro (*S. weddelliana*), and into the state of São Paulo (*S. hoehnei*).

Most of the species found in branch 57’ are from the Caribbean, northern part of South America, the Amazon or the Andes. *Syagrus amara* from the Caribbean logically falls out in branch 62 next to *S. stenopetala* (Venezuela) and *S. orinocensis* (Venezuela, Colombia), the next closest *Syagrus* species. In branch 60, *S. stratincola* and *S. inajai* co-exist in the rain forests of Suriname and French Guyana of northern South America. *Syagrus inajai*, which is found throughout much of the Amazon basin also emerges near *S. botryophora* of the Atlantic Forest, an Amazonian connection that was also resolved in the molecular analyses (Meerow 2009, 2014). A similar Amazonian/Atlantic Forest connection pops up in branch 67’ between the pre-Amazonian species of *S. vermicularis* and the Atlantic Forest species, *S. pseudococos*. This Amazonian connection shown both here and in the molecular analyses offers further evidence that the two forests were connected at one time. Finally, several Andean species emerge in branch 60’ with *S. smithii* (Colombia, Ecuador, and Peru), *S. sancona* (Venezuela to Bolivia), and *S. cardenasii* (Bolivia).

## Conclusion

As demonstrated in the figures, there is a great deal of diversity in the leaflet anatomy of the genus *Syagrus*. This key has made use of this diversity to create a valuable and easy tool for the identification of *Syagrus* species using anatomical characters found in leaflet margin cross-sections. It will especially be found to be useful when the palms have not yet produced any reproductive material and little else is known about the palm under study. It can also be used to help confirm identifications.

The key can be seen as a crude means to infer certain relationships within the genus. The anatomical key based on phenetics, not only repeated some of the same groupings or clades that were seen in the molecular analyses, but also grouped the species geographically as well. Only further, more inclusive, molecular analyses will determine if the relations found here are substantiated by the DNA sequences.
